# miR-196b-Mediated Translation Regulation of Mouse *Insulin2* via the 5′UTR

**DOI:** 10.1371/journal.pone.0101084

**Published:** 2014-07-08

**Authors:** Amaresh C. Panda, Itishri Sahu, Shardul D. Kulkarni, Jennifer L. Martindale, Kotb Abdelmohsen, Arya Vindu, Jomon Joseph, Myriam Gorospe, Vasudevan Seshadri

**Affiliations:** 1 National Centre for Cell Science, Ganeshkhind, Pune, India; 2 Laboratory of Genetics, National Institute on Aging, NIH, Baltimore, Maryland, United States of America; Korea University, Korea, Republic Of

## Abstract

The 5′ and the 3′ untranslated regions (UTR) of the insulin genes are very well conserved across species. Although microRNAs (miRNAs) are known to regulate insulin secretion process, direct regulation of insulin biosynthesis by miRNA has not been reported. Here, we show that mouse microRNA miR-196b can specifically target the 5′UTR of the long insulin2 splice isoform. Using reporter assays we show that miR-196b specifically increases the translation of the reporter protein luciferase. We further show that this translation activation is abolished when Argonaute 2 levels are knocked down after transfection with an Argonaute 2-directed siRNA. Binding of miR-196b to the target sequence in insulin 5′UTR causes the removal of HuD (a 5′UTR-associated translation inhibitor), suggesting that both miR-196b and HuD bind to the same RNA element. We present data suggesting that the RNA-binding protein HuD, which represses insulin translation, is displaced by miR-196b. Together, our findings identify a mechanism of post-transcriptional regulation of insulin biosynthesis.

## Introduction

Insulin is a small peptide hormone secreted by pancreatic β cells and is important for glucose homeostasis in mammals. Insulin expression begins at embryonic E9.5 day in the gut endoderm [1]. Insulin expression in β cells is regulated by many nutrients, but mainly by glucose. Interestingly, glucose stimulation results in insulin secretion within minutes [2] and is immediately followed by specific increase in insulin translation [3]. The 5′ and 3′un-translated regions (UTR's) of *insulin* mRNA have been shown to have a role in this translation regulation [4]; [5]. In mouse, two non-allelic genes encode for insulin and specific splice variants from these genes have also been reported [6]; [7]. Some of the splice variants have altered 5′UTR and have differential translation efficiency and hence have been implicated in diabetes [6].

MicroRNAs (miRNAs) are short (∼22-nt) regulatory RNAs that influence a number of pancreatic events, including the development of pancreatic islets and β cells [8]; [9], insulin secretion [10]-[12], insulin resistance and diabetes [13]-[15]. Normally, miRNAs target the 3′UTRs, causing degradation and/or translational repression of the target mRNA [16]; occasionally, miRNAs have also been found to activate translation through the 3′UTR [17]. In addition, bioinformatics studies have suggested the presence of a large number of potential target sites in the 5′UTRs and coding regions of mRNAs [18]. 5′UTR-targeted miRNA-mediated translation increase has been shown for miR-10a and the target ribosomal protein mRNA [19]. miRNA can increase translation by targeting the 5′UTR and 3′UTRs with or without involvement of Argonaute 2 (Ago2), a key player of the RISC (RNA-induced Silencing Complex) [19]; [20]. However, the mechanism of miRNA-mediated increase in translation is not fully understood.

In our previous study, we showed that mouse *insulin2* mRNA undergoes alternative splicing, resulting in a shorter 5′UTR splice variant *insulin2-S* lacking 12 nucleotides in the 5′UTR. The short 5′UTR splice variant constitutes 75% of the insulin2 mRNA pool, and has an increased translation efficiency [7]. In the present study, we show that miR-196b can specifically target the 5′UTR of *insulin2* mRNA (the longer 5′UTR containing a splice variant) and regulates its translation in an Ago2-dependent manner. Interestingly, miR-196b increases target gene expression without affecting *insulin2* mRNA levels but by enhancing the size of *insulin2* mRNA polysomes, indicating that miR-196b upregulates *insulin2* translation.

## Results

### Identification of miRNA that target 5′UTR of mouse insulin mRNA

*Insulin* mRNA expressed in mouse pancreas consists of a pool of transcripts containing different 5′UTRs. The variations in the 5′UTR are due to two non-allelic genes as well as alternative splicing, resulting in at least three different 5′UTRs in insulin mRNA. In light of recent reports that microRNAs can function through the 5′UTRs of target mRNAs, we explored the possibility that the insulin mRNA 5′UTR isoforms could contribute to their differential regulation via miRNA actions. miRNAs that can potentially target insulin 5′UTR were identified by MicroInspector web tool. Four miRNAs (miR-196b, miR-323-5p, miR-338-5p and miR-370) with high complementarity to seed sequences (at least 5 base pairs between nucleotide position 2–8 of the miRNA) and a free energy of less than −22 kCal/Mole were selected (Table 1) for further analysis.

**Table 1:** miRNAs with potential target sites in the 5′UTR of mouse insulin mRNA and its binding energy to mRNA.

5′UTR of mouse *insulin* mRNAs	Potential miRNAs	sequence of miRNA	Free Energy (ΔG, kcal/mol)
*Insulin1*	mmu-miR-370	gccugcugggguggaaccuggu	−27.5
	mmu-miR-468	uaugacugaugugcgugugucug	−20.4
*Insulin2*	mmu-miR-196b	uagguaguuuccuguuguuggg	−27.9
	mmu-miR-196a	uagguaguuucauguuguuggg	−21.1
*Insulin2-S*	mmu-miR-338-5p	aacaauauccuggugcugagug	−23.8
	mmu-miR-323-5p	aggugguccguggcgcguucgc	−22.4

### Mouse miR-196b activates the expression of insulin2-5′UTR reporter construct

**Figure 1:**
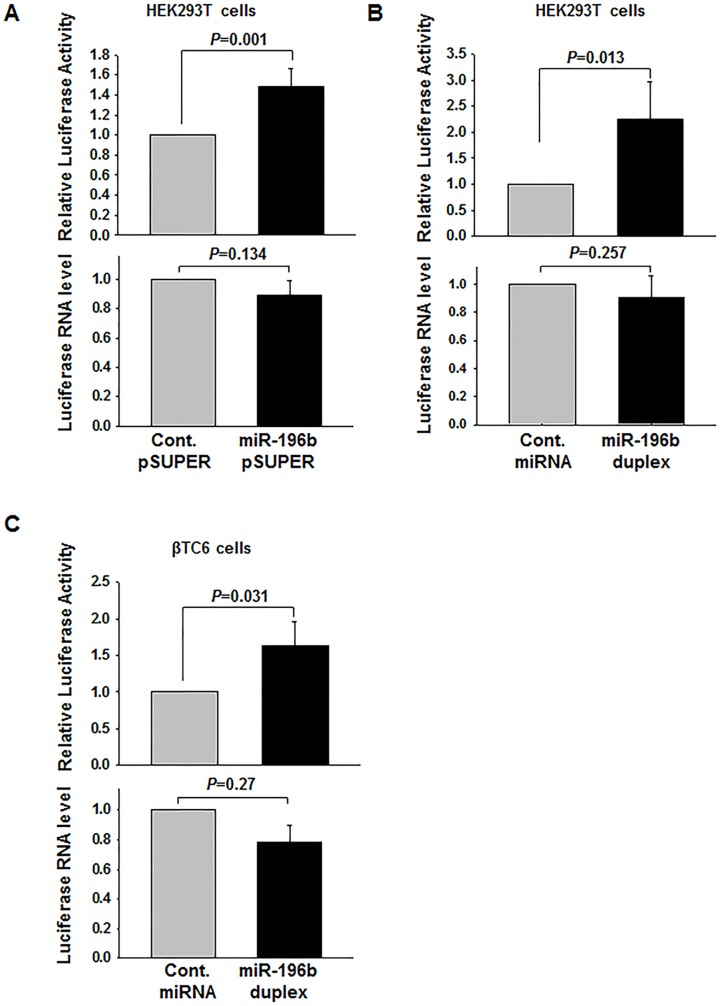
miR-196b activates insulin2-reporter expression. **(A)** HEK293T cells were co-transfected with the insulin2 reporter and with miR-196b pSUPER/cont miR pSUPER. Forty-eight hr later, firefly and Renilla luciferase activities were measured. **(B, C)** The miR-196b duplex/Control siRNA was transfected along with insulin2 reporter construct and Renilla luciferase as internal control in HEK293T (B) or βTC6 (C). The fold change in translation is shown for the insulin2 reporter, with expression levels of control miRNA-transfected cells set to 1. The relative RNA levels as assessed by RT-qPCR are indicated in the bottom panel. The graphs represent the means ± SD of 3–9 independent experiments; *P* values (Student's t-test) are indicated.

The translation regulation ability of these miRNAs was tested using a luciferase reporter system. Insulin 5′UTR corresponding to mouse *insulin1*, *insulin2* and *insulin2-S* were cloned at the 5′UTR of a luciferase reporter gene. The luciferase reporter plasmids, the control renilla plasmids, and the microRNAs were transfected into human embryonic kidney (HEK) 293T cells; 48 hours later, luciferase activity was measured. Transfection of miR-323-5p (data not shown), miR-338-5p, and miR-370 plasmids did not affect the expression of luciferase reporter from constructs containing the respective target sequence in the 5′UTR (Fig S1A–B). Additionally, transfection of miR-370 and miR-338-5p duplexes did not show any significant change in reporter activity when transfected with *insulin1* and *insulin2-S* reporters, respectively (Fig. S1C,D). Similarly, transfection of miR-370 and miR-338-5p duplexes along with insulin2 reporter also did not show any change in reporter activity when compared to control miRNA (Fig. S1E). However, transfection of miR-196b plasmid resulted in an increase in luciferase activity from constructs containing the target insulin2 5′UTR by about 50% (Fig. 1A). Luciferase mRNA levels, assessed by quantitative PCR, showed that miR-196b transfection did not result in increased luciferase mRNA levels, but actually caused a modest decrease in luciferase mRNA levels. Thus, the increase in relative luciferase activity upon miR-196b transfection was likely due to an increase in translation and not due to increased mRNA abundance. The expression of miR-196b had no effect on the other 5′UTR luciferase constructs that did not contain the target sequence in the 5′UTR.

**Figure 2:**
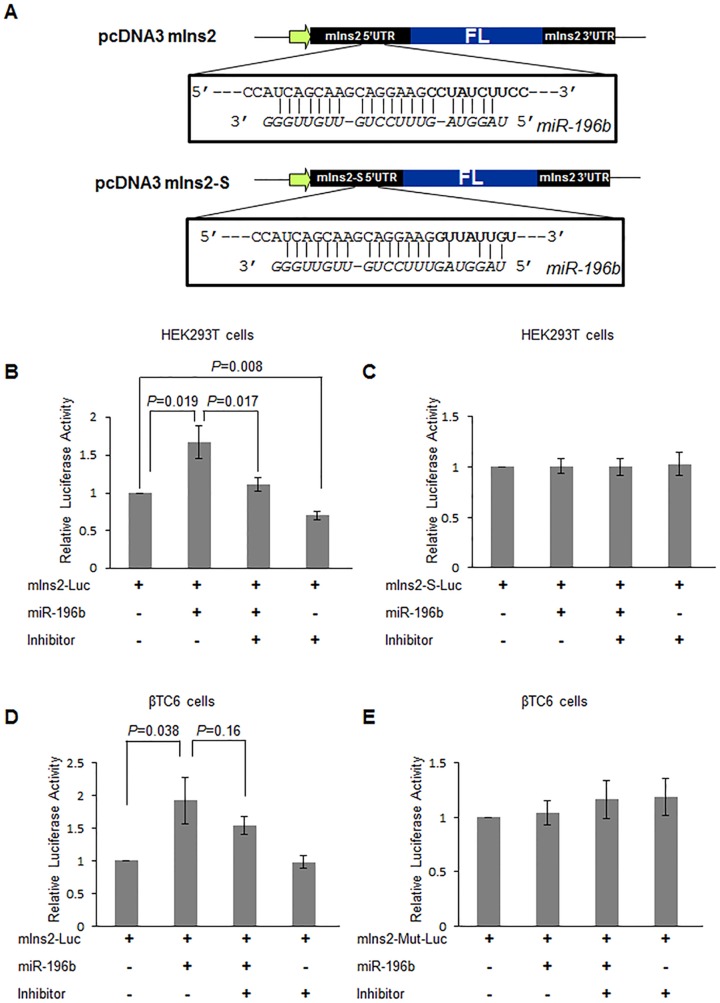
Translation activation mediated by miR-196b can be abolished by the anti miR-196b inhibitor. (**A**) Schematic representation of the reporter constructs for insulin2 and insulin2-S 5′UTR constructs. The bold letters in the RNA sequence represents the start of exon 2 of *insulin2* mRNA and the lower *italic* letters represents the miR-196b sequence. The vertical lines between the sequences denote base pairing. (**B, C**) Anti-miR-196b was introduced into cells along with reporter and the miRNA-pSuper; 48 hr later, the effect of the miR-196b inhibitor was analyzed by measuring the relative luciferase activity in HEK293T cells transfected with insulin2 reporter (B) or insulin2-S reporter (C). (**D, E**) The miR-196b inhibitor and miR-196b duplex/control miRNA were introduced into βTC6 cells before transfecting with insulin2-luciferase reporter (D) or the mutant-reporter (E); 48 hr later, the effect of miR-196b inhibitor was analysed by measuring the relative luciferase activity in the indicated treatment groups. The graphs represent the means ± SD of 3 independent experiments; *P* values (Student's t-test) are indicated.

Expression of miR-196b did not change significantly the luciferase activity derived from insulin2-luciferase in βTC6 cells, an insulin-producing cell line (Fig. S2A), possibly because of low expression levels of the miRNA and/or because of the large amount of endogenous *insulin2* mRNA already present in the cell. In order to overcome this limitation, we synthesized the duplex miR-196b RNA and co-transfected it with the insulin2-5′UTR-luciferse construct in HEK293T and βTC6 cells. A ∼140% increase in luciferase expression was observed in HEK293T cells when compared to control miRNA without any significant change in the mRNA level (Fig. 1B). Interestingly, in βTC6 cells miR-196b expression increased the relative luciferase activity of the insulin2 5′UTR-containing reporter (Fig. 1C). Surprisingly, The miR-196b target site is just at the exon1-exon2 junction of the *insulin2* mRNA which makes it very specific to this *insulin2* isoform, not the *insulin2-S* splice variant, which lacks the miR-196b seed sequence. Therefore, insulin2*-S* 5′UTR containing reporter was used as a control reporter in all further experiments (Fig. 2A). The control insulin2-S luciferase reporter did not show any significant change in the expression with miR-196b transfection in βTC6 cells (Fig. S2B). These data suggest that the underlying mechanism of the miR-196b-mediated translation regulation of the *insulin2* 5′UTR containing mRNA can occur in both insulin-producing and non-producing cells.

We assessed the specificity of the miR-196b-mediated translation regulation by using a miR-196 antagonist, antisense miR-196b. The Luc reporter plasmid was co-transfected with miR-196b or control miRNA and 2′O-methylated antisense inhibitor of miR-196b, in HEK293T cells. miR-196b inhibitor blocks the miR-196 mediated activation of insulin2-5′UTR-Luciferase translation (Fig. 2B). The control reporter without the miR-196b target site showed no significant change in expression with the inhibitor (Fig. 2C). Similar results were obtained with βTC6 cells (Fig. 2D). The mutant insulin2-reporter expression was not affected by miR-196b in βTC6 cells (Fig. 2E). These results indicate that mouse miR-196b regulates the translation of the mouse *insulin2* mRNA by targeting its 5′UTR.

### Argonaute 2 is required for miR-196b-mediated translation upregulation

**Figure 3:**
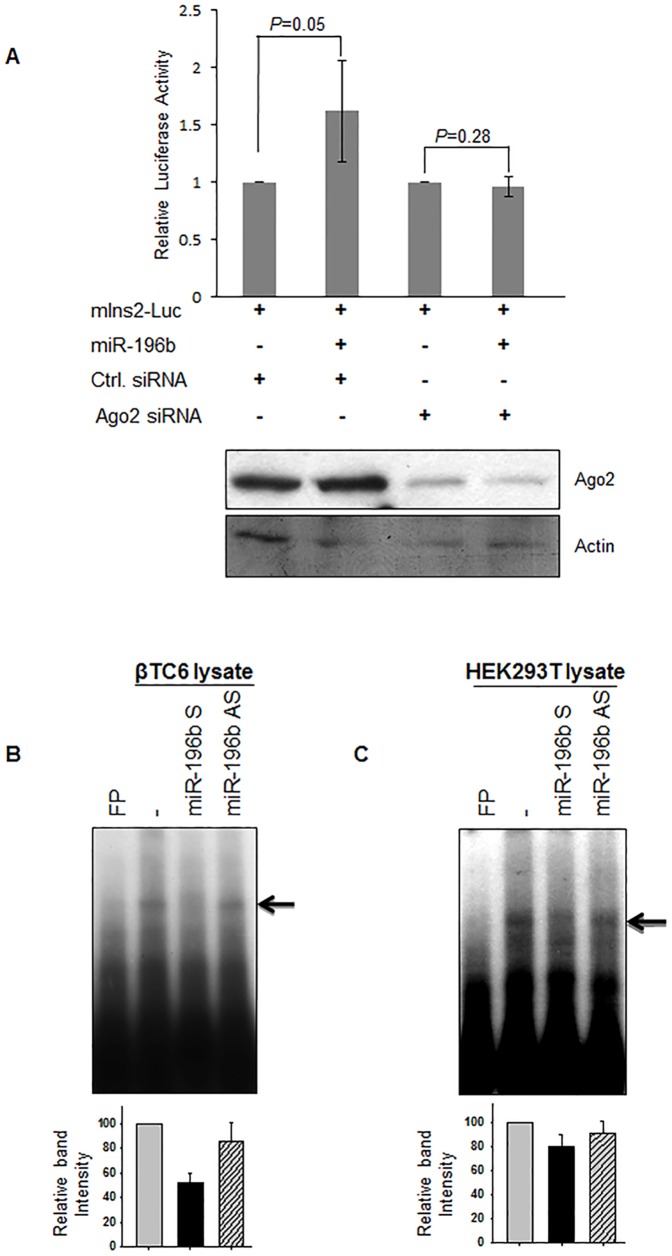
miR-196b requires Ago2 for translation activation of the target mRNA. (**A**) Reporter insulin2-Luc was cotransfected into HEK293T cells along with miR-196b, control siRNA or Ago2 siRNA. The fold change in relative luciferase activity was measured with the activity of the luciferase construct with control miRNA set to 1. *Bottom*, representative western blot to detect the levels of Ago2 and loading control β-Actin. The sample in each lane is indicated. (**B, C**) RNA-EMSA using the radiolabeled *insulin2* RNA as probe and extracts from βTC6 (B) or HEK293T (C) cells. The arrow indicates the shifted RNP complexes. The lower panel shows the RNA-protein complex intensity as measured by densitometry. The graphs represent the means ± SD of 3 independent experiments; *P* values (Student's t-test) are indicated.

Since miR-196b targets the 5′UTR and activates translation, we tested whether Ago2, an important component of the RISC, is required for translation activation mediated by this microRNA. We knocked down Ago2 using a specific siRNAs in HEK293T cells and transfected the reporter containing *insulin2* 5′UTR along with miR-196b. Ago2 siRNA reduced Ago2 expression levels by almost 70% and the miR-196b-mediated translation activation was almost completely inhibited, while the control siRNA did not show any significant alterations in the expression levels (Fig. 3A).

### miR-196b inhibits the formation of Insulin2-5′UTR-protein complexes

We have previously shown that specific factors bind to the insulin 5′UTR with differential efficiency [7]. A specific RNA-protein complex is formed with *insulin2* 5′UTR and cytoplasmic protein factors, but in case of insulin2-S, the complex formation is reduced. *In vitro* and *in vivo* translation experiments showed a correlation between the complex formation and reduced translation efficiency, suggesting that the *trans*-acting factor that associates with *insulin2*-5′UTR is likely to be a translation inhibitor. The translation efficiency of *insulin2* mRNA is the lowest among mouse *insulin* mRNAs (Fig. S3). We hypothesized that the miRNA could activate translation by interfering with the RNA-protein interactions at the 5′UTR. Therefore, we analysed the RNA-protein complex formation in the presence and absence of miRNA-196b. The miR-196b sense strand or antisense strand were incubated with the *insulin2* 5′UTR before the addition of lysate, and the RNA-protein complex formation was assessed by RNA electrophoretic mobility shift assay (REMSA). The sense strand miR-196b inhibits the complex formation while the antisense strand had no effect on the complex formation in βTC6 cells (Fig. 3B). We observed similar results using extracts from HEK293T cells (Fig. 3C). These data suggest that the binding of miR-196b disrupts the RNP (ribonucleoprotein) complex formation.

### miR-196b causes reduced association of mouse Insulin2 mRNA with HuD

**Figure 4:**
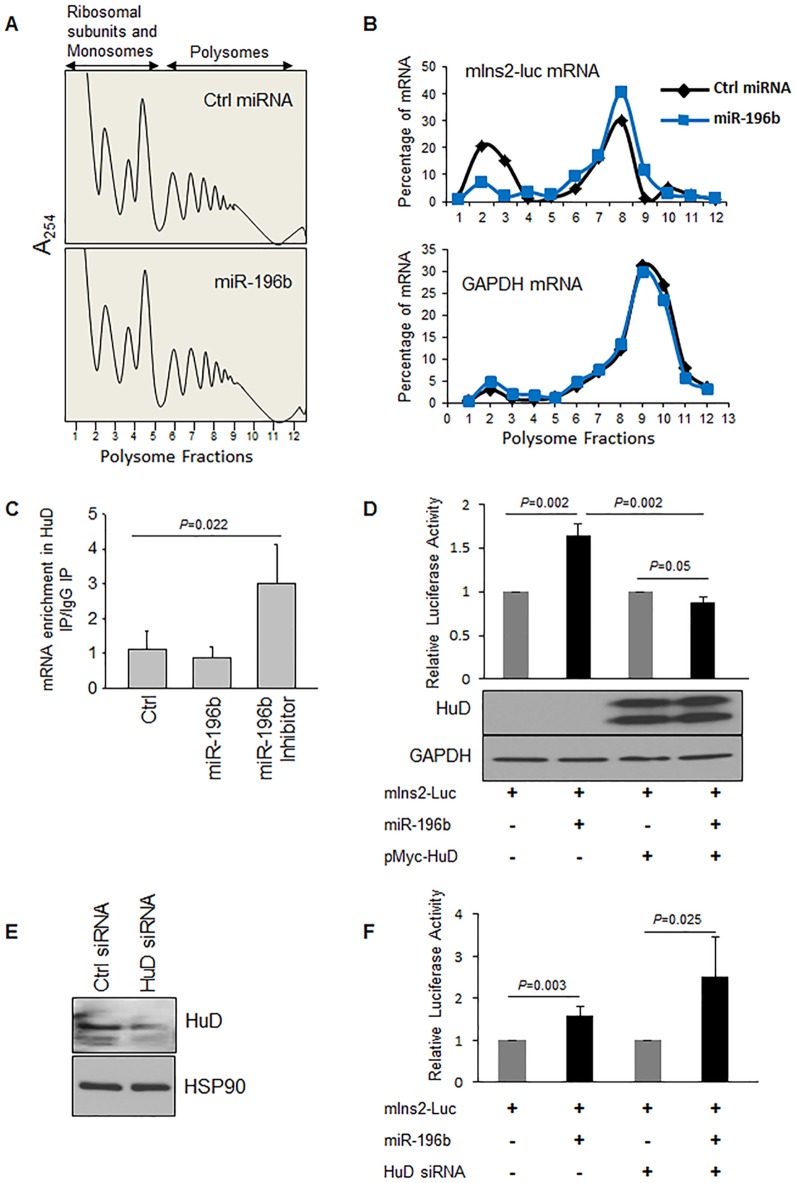
miR196b induces polysome association of Insulin2 reporter mRNA by inhibiting HuD binding. (**A**) Lysates prepared from βTC6 cells transfected with Ctrl miRNA or mature miR-196 were fractionated through sucrose gradients to generate polysome profiles. Fractions 1–5 and 6–12 were considered as non-polysome and polysome respectively. (**B**) The relative distribution of *Insulin2* reporter mRNA and *GAPDH* mRNA on polysome gradients was studied by RT-qPCR analysis of the RNA present in each of 12 gradient fractions, and represented as percentage of total mRNA. One of the representative experiments is shown here. (**C**) Interaction of HuD with *Ins2-reporter* mRNA in βTC6 cells transfected with control, mature miR-196b or miR-196b inhibitor, was studied by mRNP IP analysis using anti-HuD or control IgG antibodies. The RNA in the IP material was isolated, and *Ins2*-reporter mRNA levels were measured by RT-qPCR analysis and normalized to *PGK* mRNA levels. (**D**) Translation upregulation of insulin2 reporter with miR-196b in HEK293T cells transfected with either control or Myc-HuD plasmid. Lower panel shows the immunoblot for the over expression of Myc-HuD. (**E, F**) Forty-eight hours after transfection of βTC6 cells with Ctrl siRNA or HuD siRNA, HuD silencing was assessed by western blot analysis (E). Luciferase reporter with *insulin2* 5′UTR along with miR-196b or control miRNA duplex was introduced into βTC6 cells expressing normal or reduced HuD levels (F). The fold change in relative luciferase activity was measured with the activity of the luciferase construct with control miRNA set to 1. The graphs in (C,D,F) represent the means ± SD of 3-9 independent experiments; *P* values (Student's t-test) are indicated.

To study the effect of miR-196b on insulin translation, we analyzed the association of Insulin2 reporter with the cell′s polysomes in Ctrl siRNA- or miR-196b transfected βTC6 cells. miR-196b did not affect the global translation profile of βTC6 cells (Fig.4A). RT-qPCR analysis of polysomal fractions show an increased association of the insulin 2 reporter with the polysome upon miR-196b treatment, suggesting that miR-196b promotes the translation of Insulin2 in βTC6 cells (Fig.4B). However, we did not see specific change in polysome association of endogenous mouse *insulin2* mRNA following miR-196b transfection (Fig.S4), perhaps because the vast abundance of endogenous *insulin* mRNA.

Recently, Lee et al. have shown that the RNA-binding protein HuD bind to insulin 5′UTR and repressed the translation of insulin mRNA [21]. As miR-196b also targets the 5′UTR of *insulin2* mRNA, we sought to determine if there is any interplay between HuD and miR-196 in controlling Insulin2 translation. Following transfection of control RNA, miR-196b miRNA or miR-196b-as inhibitor into βTC6 cells along with the Insulin2 reporter, the interaction of HuD with the reporter mRNA was assessed by ribonucleoprotein immunoprecipitation (RIP) analysis followed by detection of the reporter RNA in the IP material. Interestingly, we found increased association of HuD with the insulin 5′UTR reporter after antagonizing miR-196b function (miR-196b-as group), while expression of miR-196b modestly decreased the association of the insulin 5′UTR reporter mRNA with HuD. These results suggest that HuD and miR-196b might compete for binding to the 5′UTR of *Ins2* mRNA (Fig. 4C). We also assessed the effect of the miR-196b expression on the association of HuD with endogenous insulin mRNA and find similar trend, although to a lesser degree (Fig. S5).

### miR-196b competes with HuD for binding to 5′UTR of Insulin2 mRNA

To analyse the functional interplay between miR-196b and HuD upon insulin 2 translation we followed the gain of function and loss of function experiments. The over expression of HuD in HEK293T cells along with the insulin2 reporter and miR-196b abolished the miR-196b mediated translation up regulation (Fig. 4D). This finding suggests that HuD/miR-196b compete for binding to 5′UTR of *insulin2* mRNA. Further, we silenced HuD in βTC6 cells and studied the effect of HuD knock down on translation upregulation by miR-196b (Fig. 4E). Upon HuD silencing, we observed even higher insulin2 translation as compared with miR-196b overexpression alone (Fig. 4F).

### miR-196b is expressed in mouse βTC6 cells and embryonic pancreas

**Figure 5:**
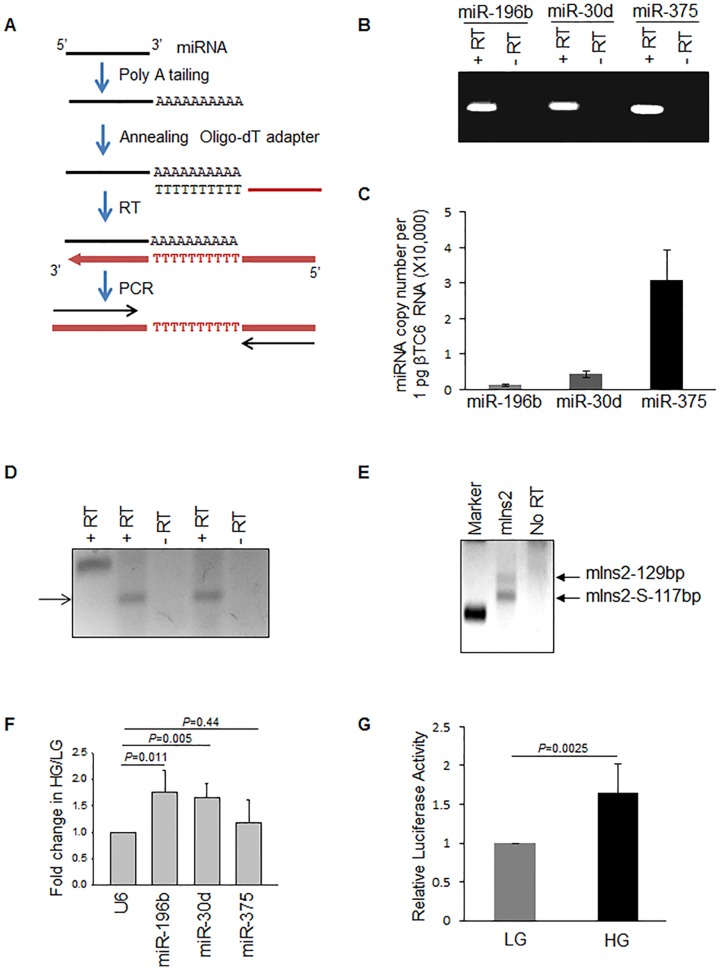
Expression of miR-196b. (**A**) Schematic of the method used for miRNA RT-PCR using QuantiMiR kit (SBI). (**B**) The cDNA for miRNA RT-PCR was prepared from 1 µg of total RNA from βTC6 cells using QuantiMiR RT kit as per the manufacturer protocol. The upper panel shows the average absolute values calculated from Ct values of RT-qPCR of miRNAs amplified using universal reverse primer from the QuantiMiR kit and specific forward primers for respective miRNAs. The lower panel shows the miRNA RT-PCR or No RT-PCR products were resolved on a 3.5% agarose gel. (**C**) Comparison of miR-196b, miR-30d, and miR-375 expression from RT-qPCR data of βTC6 total. Copy numbers per 1 pg of total RNA were calculated using standard curve based on known amount of miRNA. (**D**) cDNA for miR-196b was prepared by miR-196b-RT stem-loop primer and amplified with specific primers (primers 18 and 20, Table S1). The cDNA for miR-375 was prepared by miR-375-RT stem-loop primer and amplified with specific primers (primers 19 and 20, Table S1) from e14.5 day pancreas and the PCR product was resolved on a 3.5% agarose gel. (**E**) *Insulin2* RT-PCR detection using gene-specific primer (primers 1 and 2, Table S1) using RNA isolated from e14.5 day mouse pancreas. (**F**) The change in expression of various miRNAs in high glucose treated βTC6 cells using QuantiMiR RT-qPCR kit. (**G**) Expression of insulin2 reporter in high glucose treated βTC6 cells normalized to renilla expression. In (C,F,G) the graphs represent means ± SD of 3-8 independent experiments; P values (Student's t-test) are indicated.

The expression levels of various miRNAs were analysed in mouse βTC6 cells. miR-196b, miR-338-5p, miR-370 and miR-375 were previously reported to be expressed in adult mouse pancreas [22]. We detected miR-196, miR-30d and miR-375 in βTC6 cells using QuantiMir RT-PCR kit (Fig. 5A-B). We then calculated the copy number of the above miRNAs using known amounts of miRNA using the QuantiMiR assay system by RT-qPCR (Fig. 5C). This result shows the relatively lower expression of miR-196b in βTC6 cells as compared to other two well-characterized miRNAs. We also detected miR-196b expression in mouse embryonic pancreas by using the stem-loop primer RT-PCR method (Experimental Methods). In mouse embryonic pancreas at day e14.5, specific PCR products were detected corresponding to miR-196b and miR-375 (Fig. 5D), and isoforms of *insulin2* mRNA such as *insulin2* and *insulin2-S* (Fig. 5E). These data indicate that during embryonic development miR-196b and *insulin2* were expressed together in the same tissue.

### Glucose increases the miR-196b expression in βTC6 cells

Recent reports suggested that miRNAs are regulated by glucose in MIN6 cells. In order to analyse the effect of glucose on the expression on miR-196b in βTC6 cells, we incubated βTC6 cells in the absence of glucose or in the presence of 25 mM glucose for 16 h, and prepared total RNA. Analysis of the miRNA cDNA (using QuantiMiR kit) indicated that miR-196b along with miR-30D was upregulated in the presence of high glucose, whereas miR-375 level did not change significantly (Fig. 5F). The insulin2 reporter expression also increased under these conditions, suggesting a potential positive influence of the heightened miR-196b levels (Fig. 5G). These results suggest that increased miR-196b expression in response to glucose may be an additional mechanism for glucose-stimulated insulin synthesis.

## Discussion

miRNAs regulate the expression of an estimated one-third of genes in mammals [23]; [24]. miRNAs typically target the 3′UTR of the mRNA and suppress its expression by inhibiting translation and/or by degrading the target mRNA [16]; [25]. Recently, several miRNAs have been shown to target other regions in the mRNA and some of these miRNAs have also been shown to increase the expression of target genes. In pancreatic β cells, several miRNAs and RNA binding proteins have been reported to regulate the glucose-induced insulin expression [26]; [27]. Here, we report the role of mouse miR-196b in the regulation of *insulin2* mRNA translation. miR-196b exists in the HOX gene cluster and regulates HOXB8 gene by targeting the 3′UTR expressed in mouse embryonic stages [25] as well as in adult pancreas [22]. We show that miR-196b is expressed at day e14.5 in the embryonic pancreas and propose that it could be involved in the regulation of translation of insulin genes specifically during embryonic development. This increased translation of insulin could be important since embryonic pancreas needs to maximize the production of insulin for organogenesis and growth. We believe that miR-196b-mediated translation upregulation of embryonic insulin is an adaptation to this specific need for increased insulin.

**Figure 6:**
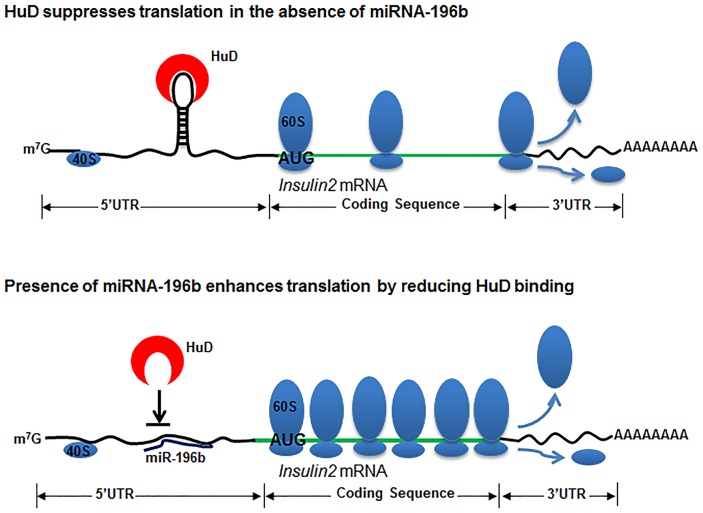
Mechanism of miR-196b action. The miR-196b target site is at the 5′UTR stem loop structure of the *insulin2* mRNA. Targeting of miR-196b to the stem-loop region of the *insulin2* mRNA disrupts the secondary structure and prevents binding of the translational inhibitor, resulting in the activation of insulin translation.

miR-196b targets the 5′UTR of mouse *insulin2* mRNA and we hypothesize that the miRNA mediated up-regulation is due to reduced association of HuD to insulin 5′UTR (Fig. 6). The decreased binding of HuD is probably due to altered stem-loop structure of the 5′UTR or due to competition with miRNA for the binding to mRNA. These results suggest a coordinated regulation of translation by miRNA and UTR-binding proteins, similar to those observed in case of miR-466I [28]. Ago2 is an important player in miRNA-mediated regulation of gene expression; our results indicate that the miR-196b-mediated activation of insulin expression also requires Ago2, suggesting that the miRNA-196b-mediated displacement of HuD might involve Ago2, although the exact mechanism of the translation regulation is still unknown. This regulation is similar to the regulation observed for mRNA of some of the ribosomal genes that contain the TOP element in the 5′UTR. These mRNAs are positively regulated by miR-10b and negatively regulated by binding of specific factors to the 5′UTR of the mRNA [29].

The physiological relevance of this regulation is still unclear, as *insulin2* isoform contributes only about 13% of total *Insulin* mRNA and is poorly translated when compared to the mouse *Insulin1* or the *insulin2-S* isoform [7]. Thus, under normal physiological conditions, the protein product from this mRNA isoform is less than 10% of the total insulin produced (Fig. S3). But this regulation could be important in embryonic development where it can significantly increase the insulin production from a reduced pool of insulin mRNA transcripts.

The competition between HuD and miR-196 for binding to *insulin2* mRNA may explain the mechanism of translation upregulation by miR-196b. The requirement of Ago2 for such an upregulation suggests the involvement of RISC complex in this activity and not simply an antisense RNA activity. Further, regulation of miR-196b expression by glucose suggests an additional level of regulation of insulin biosynthesis by a novel mechanism that could play an important role in the case of diabetes.

In summary, the present study demonstrates for the first time that a microRNA can target a specific splice variant of the insulin mRNA and can promote its translation. We propose that miR-196b interaction with insulin mRNA disrupts the interaction of HuD to the mRNA thereby resulting in translation activation.

## Experimental Methods

### miRNA search

Potential miRNAs that can target mouse insulin mRNAs were identified by using the MicroInspector web tool (http://bioinfo.uni-plovdiv.bg/microinspector/). This search engine predicts the miRNAs based on the seed sequence complementarity and binding energy (ΔG) of miRNA-mRNA duplex.

### Generation of plasmid constructs

Double-stranded oligomers representing the pre-miR-196b with Hind III (5′) and Sal1 (3′) site (Table S1) were ligated to the pSUPER vector (Oligoengine). The insulin2-5′UTR luciferase and insulin2-S-5′UTR luciferase pcDNA3 constructs were generated as described previously [7].

### Pancreas isolation and cell culture

These procedures were carried out in strict accordance with the recommendations of Committee for the Purpose of Control and Supervision of Experiments on Animals (CPCSEA), Ministry of Environment and Forest, Government of India. The protocol was approved by the Institutional Animal Ethics Committee (IAEC) of National Centre for Cell Science (protocol Number B-192), and all efforts were made to minimize suffering of the experimental animals. BALB/c mice (6-8-weeks old) were used for pancreas isolation. The embryos from BALB/c mice were dissected from pregnant mothers at day e14.5, and the embryonic pancreas was dissected and cut off from the surrounding tissue. Adult pancreatic islets were prepared by ficoll gradient method, as described [30]. RNA was prepared using Trizol (Invitrogen, Carlsbad, CA).

HEK293T and βTC6 cells were maintained in DMEM (Sigma-Aldrich) supplemented with 10% and 15% foetal bovine serum (FBS), respectively, along with 100 µg/ml penicillin and 100 U/ml streptomycin at 37°C in a 5% CO_2_ atmosphere.

### Transfection, reporter assay and RNA quantitation

HEK293T or βTC6 cells were cultured in 24-well plates and transfected with various plasmids/RNA using Lipofectamine-2000 (Invitrogen). Ten ng of pRL-Tk (Promega) was included in all the transfection reaction to serve as transfection control. Fifty ng of luciferase reporter containing various insulin 5′UTRs, 500 ng of miR-196b-pSUPER or empty vector were transfected; 48 hr later, the cells were harvested and luciferase activity was measured with the DLR kit (Promega). The luciferase activity was normalized with the renilla expression levels.

Biotin-miR-196b RNA duplex was prepared by annealing both strands (synthesized by IDT, USA) in annealing buffer (30 mM HEPES-KOH pH 7.4, 2 mM MgCl_2_, 100 mM KCl, 50 mM NH_4_CH_3_COOH) at a final concentration of 20 µM. miR-196 or control duplexes were transfected at 40 nM concentration for 4 hr in the presence of Opti-MEM without serum and then substituted with complete medium with serum for further 2 hr before transfecting with the reporter plasmids. Unlabeled mature miR-196b duplex RNA was obtained from QIAGEN. For the miRNA inhibitor experiment, 100 pmoles of miR-196b inhibitor (5′CCCAACAACAGGAAACUACCUA3′-2′O-methyl anti-miR-196b, IDT) was transfected along with the duplex miRNA 6 hr before prior to reporter miRNA transfection. For siRNA studies, the control siRNA and the Ago2 siRNA (target sequence: GCAGGACAAAGATGTATTA, Dharmacon, USA) were transfected and recovered for 24 hr before transfection with the luciferase reporter and the miRNA constructs.

Myc-HuD plasmid (500 ng) was transfected to HEK29T cells before the transfection of insulin2 reporter and miR-196. HuD was silenced in βTC6 cells by sequential transfection of HuD siRNA (Santa Cruz), 24 hr apart, followed by the reporter and miR-196b transfection and 48 hr later the reporter expression was measured.

RNA from the transfected cells was prepared using TRIzol and the luciferase mRNA levels were assessed by RT-PCR and normalised to human beta actin or mouse GAPDH mRNA levels by quantitative PCR Assay (ABI). TaqMan qPCR for luciferase was performed using oligos 21 and 22 as primers, and 23 as probe (Table S1).

### RT-PCR analysis of miRNA and insulin mRNA

First-strand synthesis was performed from 200 ng of total RNA from embryonic pancreas using the miR-196b-RT stem-loop primer and the ImProm II reverse transcriptase kit (Promega) as described previously [31]. The RT-PCR was performed with a common reverse primer 20, and forward primers 18 and 19 for miR-196b and miR-375, respectively (Table S1) (95°C for 5 min, followed by 5 cycles of 95°C for 15 s, 36°C for 30 s and 72°C for 5 s, followed by 40 cycles of 95°C for 15 s, 50°C for 30 s and 72°C for 5 s).

For detecting miRNA in βTC6 cells, 1 µg of TRizol prepared total RNA was used for cDNA synthesis using the QuantiMir cDNA Kit (System Biosciences, Mountain View, CA) following the manufacturer's protocol. miRBase database (http://www.mirbase.org) was used to make the forward primers for RT-qPCR of the miRNAs. A universal reverse primer was supplied by the kit. The real-time PCR was performed with primers specific to the miRNA and the universal reverse primer using the manufacture's protocol.

### Immunoblotting and RNA-EMSA

HEK293T or βTC6 cells were lysed in a ice-cold lysis buffer (50 mM Tris-HCl, pH 7.5, 50 mM NaCl, 1 mM DTT 1 mM PMSF and protease inhibitor) by 10 strokes of Dounce homogenizer and the lysate was cleared by centrifugation at 20,000g for 20 minute at 4°C. Labelled *insulin2* 5′UTR was *in vitro* transcribed from the annealed double stranded oligomer using T7 RNA polymerase (Ambion) in presence of 50 µCi [α-^32^P] UTP. For EMSA reactions, 20000 cpm probe was heated at 65°C for 3 min with 10 pmoles of miR-196b sense or antisense RNA and quick-chilled on ice for 10 minute with gel shift buffer (5 mM Tris (pH 7.5), 15 mM KCl, 5 mM MgCl_2_, 0.25 mM DTT, 40 U of RNasin, and 10% glycerol) followed by the addition of 5 µg of the extract and further incubated on ice for 30 minutes. The RNP complex was analyzed by 6% PAGE, as described previously [7]. The Ago2 siRNA/Cont siRNA lysates were resolved on a 10% SDS-PAGE and proteins were transferred on to PVDF membrane. The membrane was probed with anti-Ago2 antibody (Cell Signalling) and β-Actin (Abcam) and detected by ECL™ Advance (Amersham). HuD overexpression or HuD siRNA samples were resolved in 4-20% TGX gels (BioRad) and transferred to nitrocellulose membrane. The membranes were probed with HuD, GAPDH or HSP90 antibodies from Santa Cruz.

### Polysome Analysis

βTC6 cells were transfected with insulin2-reporter along with Ctrl miRNA or miR-196b for 48hr before it was taken for Polysome fractions. The cells were preincubated with cycloheximide (Sigma; 100 µg/ml for 15 min), and cytoplasmic lysates were fractionated into 12 fractions through 15%–60% linear sucrose gradients using ultracentrifuge. RNA was prepared from the collected fractions using TRIzol (Invitrogen) and followed by RT-qPCR analysis of reporter mRNA and *GAPDH* mRNA [21].

### Endogenous HuD-mRNA complex immunoprecipitation

To analyze the association of HuD to endogenous mRNAs in βTC6 cells immunoprecipitation (IP) of RNP complexes were performed as described previously [32]. Briefly the βTC6 cells were lysed in 20 mM Tris-HCl at pH 7.5, 100 mM KCl, 5 mM MgCl_2_, and 0.5% NP-40 for 10 min on ice and cleared by centrifugation at 15,000 × g for 10 min at 4°C. The lysate was incubated with protein-A Dynabeads beads coated with antibodies recognizing HuD or control IgG (Santa Cruz Biotechnology) for 2 hr at 4°C. The beads were washed thrice with ice cold NT2 buffer (50 mM Tris-HCl [pH 7.5], 150 mM NaCl, 1 mM MgCl_2_, and 0.05% NP-40) followed by DNase treatment with 20 units of DNase I for 15 min at 37°C to remove the DNA. The samples are then incubated with 0.5 mg/ml Proteinase K supplemented with 0.1% SDS/ for 15 min at 55°C to digest the proteins. For analysis of individual mRNAs the RNA from the IP samples were prepared by phenol-chloroform and used for qRT-PCR.

## Supporting Information

Figure S1 (TIF)

Figure S2 (TIF)

Figure S3 (TIF)

Figure S4 (TIF)

Figure S5 (TIF)

Table S1 (DOC)
