# Supplementary figures and images for: miR-196b-Mediated Translation Regulation of Mouse Insulin2 via the 5′UTR
Source: PLoS One. 2014 Jul 8;9(7):e101084. doi: 10.1371/journal.pone.0101084 (PMC4086887; doi:10.1371/journal.pone.0101084)

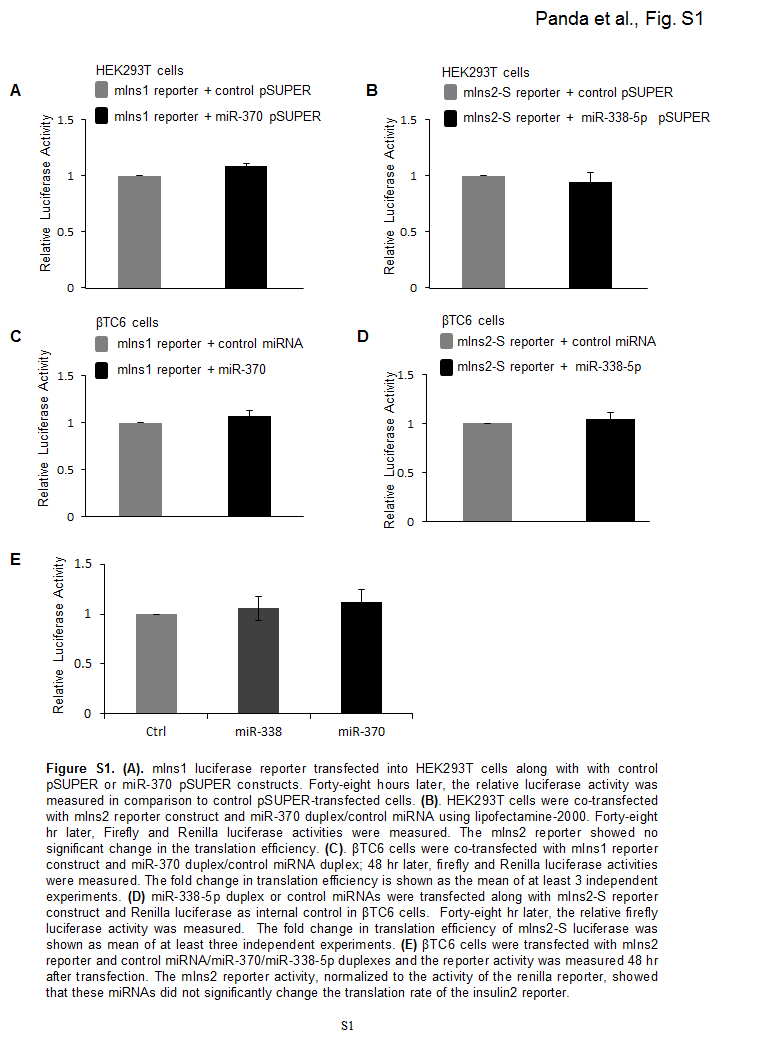

Supplement: Figure S1 — (TIF) [file pone.0101084.s001.tif]

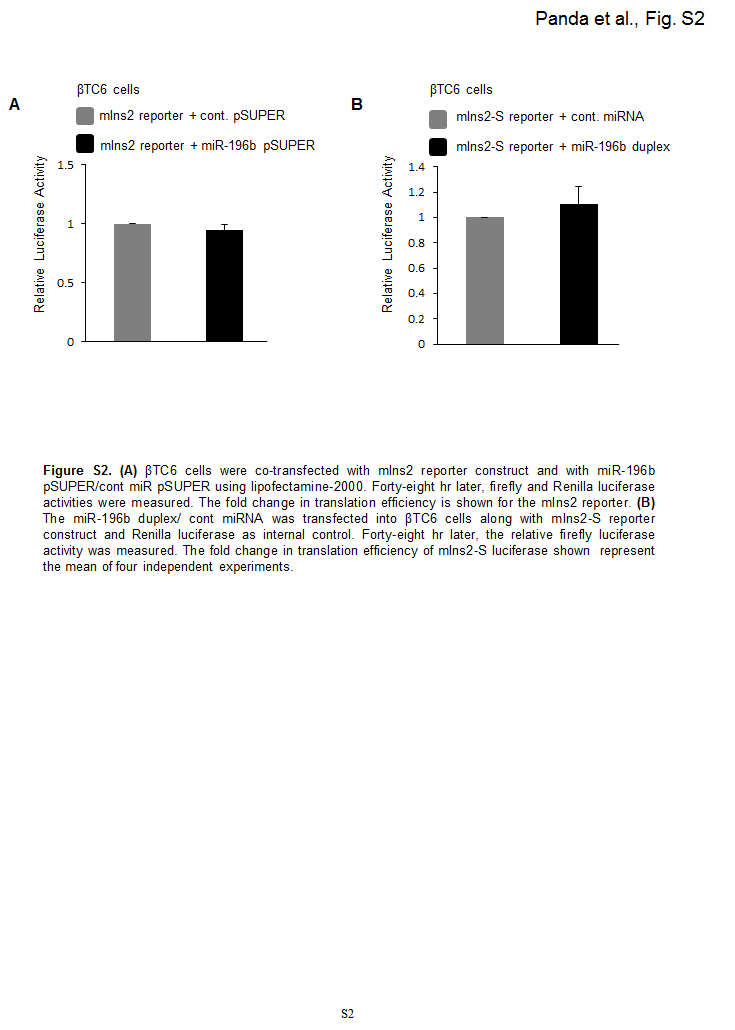

Supplement: Figure S2 — (TIF) [file pone.0101084.s002.tif]

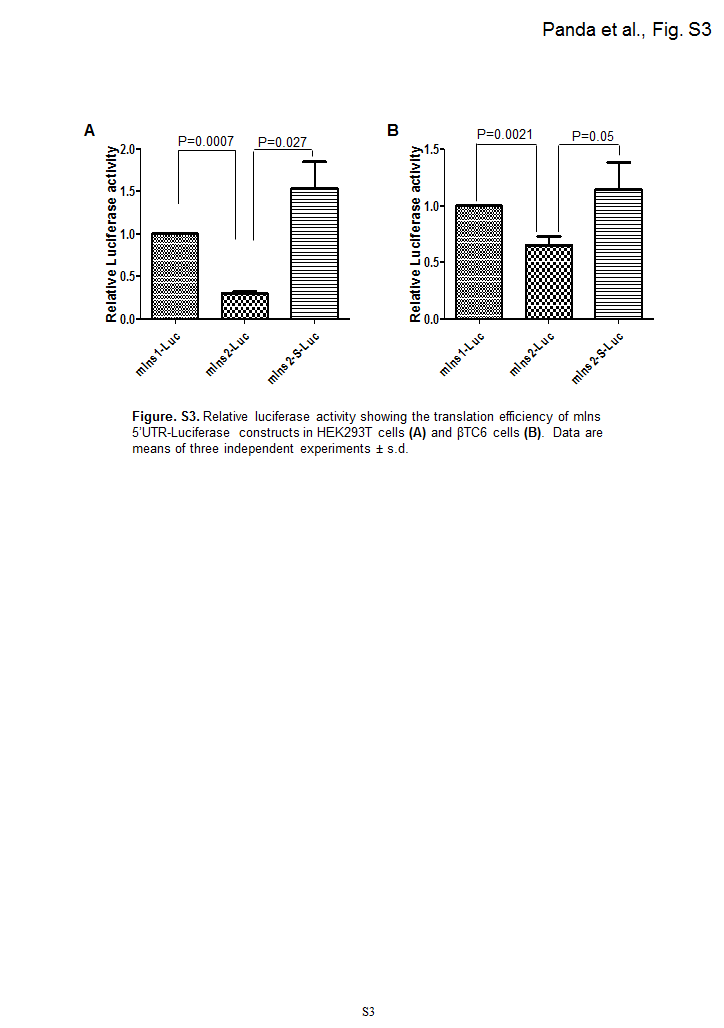

Supplement: Figure S3 — (TIF) [file pone.0101084.s003.tif]

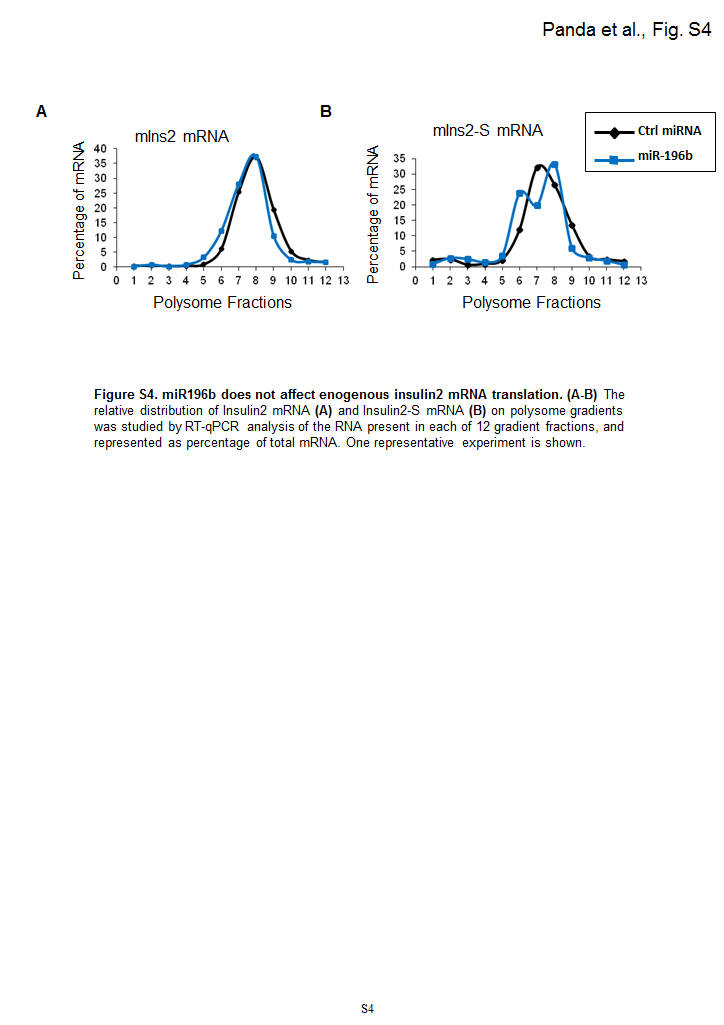

Supplement: Figure S4 — (TIF) [file pone.0101084.s004.tif]

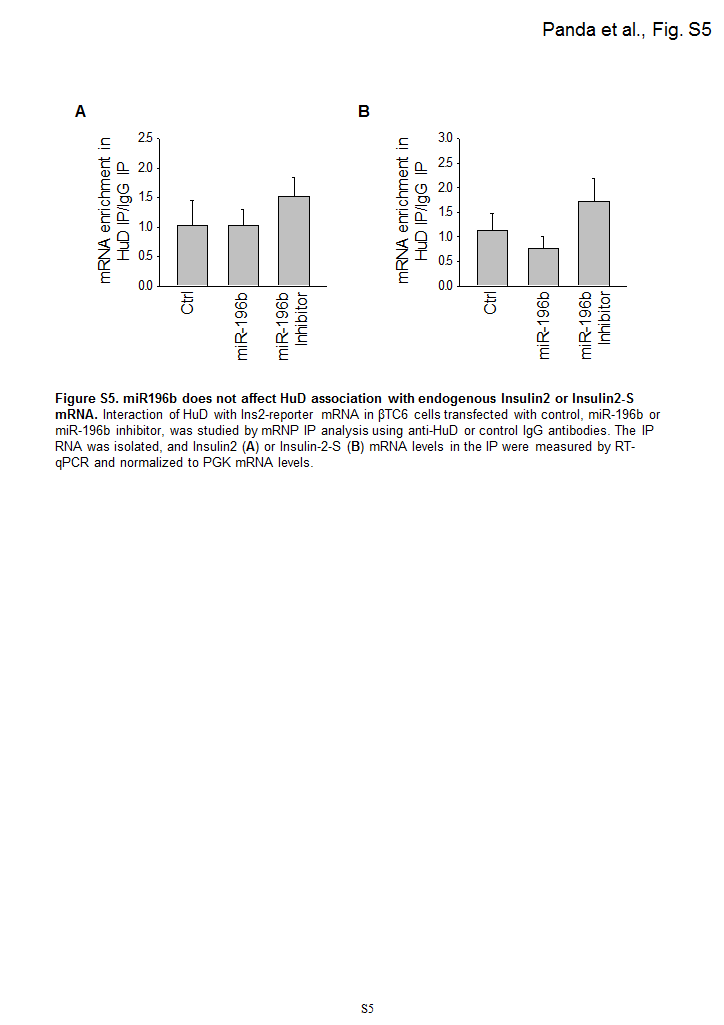

Supplement: Figure S5 — (TIF) [file pone.0101084.s005.tif]
