# Supplementary material for: miR-196b-Mediated Translation Regulation of Mouse Insulin2 via the 5′UTR
Source: PLoS One. 2014 Jul 8;9(7):e101084. doi: 10.1371/journal.pone.0101084 (PMC4086887; doi:10.1371/journal.pone.0101084)
Supplement: Table S1 — (DOC) [file pone.0101084.s006.doc]

**Supplementary Table S1:** The sequences of DNA oligonucleotides used in the study.

| **Primer No.** | **Sequence(5’ to 3’)** | **Description** |
| --- | --- | --- |
| **1** | AAGTGATCCGCTACAATCAAAA | Insulin2-SPV-F |
| **2** | GGGTGGGACTCCCAGAGGAAG | Insulin2-SPV-R |
| **3** | GTTGAAACAATAACCTGGAAGATAGGCTTCCTGCTTGCTGATGGTTTTTGATTGTAGCGGATCACTTAGGGCT**CCCTATAGTGAGTCGTATTAGCAT** | Insulin2-T7 sequence |
| **4** | GTTGAAACAATAACCTTCCTGCTTGCTGATGGTTTTTGATTGTAGCGGATCACTTAGGGCT**CCCTATAGTGAGTCGTATTAGCAT** | Insulin2-S-T7 sequence |
| **5** | ATGCTAATACGACTCACTATAGGG | T7 sequence |
| **6** | CATTCCGGATACTGCGATTT | RT Luc-F |
| **7** | GCAACGCACTTTGAATTTTG | RT Luc-R |
| **8** | **AGCTT**agccctaagtgatccgctacaatcaaaaaccatcagcaagcaggaagcctatcttccaggttattgtttcaac**g** | Insulin2-5’UTR-sense |
| **9** | **GATCC**GTTGAAACAATAACCTGGAAGATAGGCTTCCTGCTTGCTGATGGTTTTTGATTGTAGCGGATCACTTAGGGCT**a** | Insulin2-5’UTR-antisense |
| **10** | **AGCTT**agccctaagtgatccgctacaatcaaaaaccatcagcaagcaggaaggttattgtttcaac**g** | Insulin2-S-5’UTR-sense |
| **11** | **GATCC**GTTGAAACAATAACCTTCCTGCTTGCTGATGGTTTTTGATTGTAGCGGATCACTTAGGGCT**a** | Insulin2-S-5’UTR-antisense |
| **12** | **GATCCC**AACTGGTCGGTGATTTAGGTAGTTTCCTGTTGTTGGGATCCACCTTTCTCTCGACAGCACGACACTGCCTTCATTACTTCAGTTG**TTTTTG** | miR-196b-S-sequence |
| **13** | **TCGACAAAAA**CAACTGAAGTAATGAAGGCAGTGTCGTGCTGTCGAGAGAAAGGTGGATCCCAACAACAGGAAACTACCTAAATCACCGACCAGTT**GG** | miR-196b-AS sequence |
| **14** | **GATCCC**CACAGGCCGTCCTCCCCAACAATATCCTGGTGCTGAGTGGGTGCACAGTGACTCCAGCATCAGTGATTTTGTTGAAGAGGGCAGCTGCCA**TTTTTG** | miR-338-S- sequence |
| **15** | **TCGACAAAAA**TGGCAGCTGCCCTCTTCAACAAAATCACTGATGCTGGAGTCACTGTGCACCCACTCAGCACCAGGATATTGTTGGGGAGGACGGCCTGTG**GG** | miR-338-AS- sequence |
| **16** | GTCGTATCCAGTGCAGGGTCCGAGGTATTCGCACTGGATACGAC**CCCAAC** | miR-196b-stem-loop RT primer |
| **17** | GTCGTATCCAGTGCAGGGTCCGAGGTATTCGCACTGGATACGAC**TCACGC** | miR-375-stem-loop RT primer |
| **18** | CGCGTAGGTAGTTTCCTGTT | miR-196b-SL-RT-F |
| **19** | ATGCTTTGTTCGTTCGGCTC | miR-375-SL-RT-F |
| **20** | GTGCAGGGTCCGAGGT | Universal miR-RT-R |
| **21** | AGCCCTAAGTGATCCGCTACAA | Insulin2-TaqMan-F |
| **22** | GTTGCTCTCCAGCGGTTCC | Luc-5’SP |
| **23** | HEX-ATCAGCAAGCAGGAAGCCTATCTTCCAGGTTA- BHQ-2™ | Insulin2-TaqMan Probe |
| **24** | CCTTATGCAGTTGCTCTCCAGC | Luc5'-RT-R |
| **25** | CATCAGCAAGCAGGAAGCCTATC | Insulin2-F |
| **26** | CCCACACACCAGGTAGAGAG | Insulin2-R |
| **27** | CAGCAAGCAGGAAGGTTATTGT | Insulin2-S-F |
| **28** | ACCAGGTGGGAACCACAAAG | Insulin2-S-R |
| **29** | TAGGTAGTTTCCTGTTGTTGGG | miR-196b-RT-F |
| 30 | TGTAAACATCCCCGACTGGAAG | miR-30d-RT-F |
| **31** | TTTGTTCGTTCGGCTCGCGTGA | miR-375-RT-F |
| **32** | **AGCTT**AGCCCTAAGTGATCCGCTACAATCAAAAACCATCAGCAAGCAGGAAGCCTCTATTCCAGGTTATTGTTTCAAC**G** | Insulin2-5’UTR-mut sense |
| **33** | **GATCC**GTTGAAACAATAACCTGGAATAGAGGCTTCCTGCTTGCTGATGGTTTTTGATTGTAGCGGATCACTTAGGGCT**A** | Insulin2-5’UTR-mut antisense |
| **34** | CAAGGCTTTGGAGAGTCCAG | PGK-RT-F |
| **35** | TGTGCCAATCTCCATGTTGT | PGK-RT-R |
